# The prevention of respiratory syncytial virus infection

**DOI:** 10.1186/1824-7288-40-S2-A35

**Published:** 2014-10-09

**Authors:** L Bollani, M Pozzi

**Affiliations:** 1 Neonatal Intensive Care Unit, Fondazione IRCCS Policlinico San Matteo, Pavia, Italy

## 

Respiratory syncytial virus (RSV) is the most significant cause of acute respiratory tract infections in infants and young children worldwide. RSV accounts for approximately 70% of hospitalizations for bronchiolitis and 40% of pneumonia among infants <1 year of life.

RSV infection seems to be associated with recurrent wheezing during the first decade of life and impaired respiratory health-related quality of life in adults [1].

Universal prevention of RSV infection is based on environmental prophylaxis aimed at minimizing the spread of the virus good hand hygiene in the home, and limiting direct contact of high-risk children with other children and adults with respiratory tract infections. Exposure to tobacco smoke should be avoid in family with infants, breastfeeding should be encouraged. Pharmacological prophylaxis is based on the administration of palivizumab (Synagis ®, MedImmune) during the epidemic period to the children at risk [2].

Palivizumab is a humanized monoclonal antibody directed to an epitope in the A antigenic site of F protein of RSV. It is designed to provide passive immunity against RSV and thereby prevent or reduce the severity of RSV infection [2].

The aim of this work is to provide Italian neonatologists shared recommendations regarding palivizumab use in premature and other at-risk infants in the light of new emerging evidence.

The peak incidence of severe RSV disease occurs between 2 and 3 months of age. The risk of serious RSV illness is highest among preterm neonates, children with chronic lung disease, congenital heart disease [3,4].

The children with the above mentioned clinical conditions, particularly in cases of hospitalization, are more likely to require admission to an intensive care unit and need mechanical ventilation. In addition they have high rates of re-hospitalization for lower respiratory tract infections [5,6].

Therefore, all these categories of infants are likely to benefit from prophylaxis, and have been included in the recommendations. Specific recommendations are provided according to gestational age at birth

RSV infections occur most frequently during the period between October-March.

According to this observation, prophylaxis with Palivizumab is indicated in this 5-6 months long seasonal window of RSV infection.

The duration of prophylaxis (up to one year or up to two years of life during the seasonal period) depends on the underlying condition.

Palivizumab is clinically effective; however, the cost is very high. In our opinion strict criteria for patient selection and reduced drug costs would improve the cost-effectiveness of the prophylaxis [7].
